# Validation of quantitative gait analysis systems for Parkinson’s disease for use in supervised and unsupervised environments

**DOI:** 10.1186/s12883-021-02354-x

**Published:** 2021-08-28

**Authors:** Sara Alberto, Sílvia Cabral, João Proença, Filipa Pona-Ferreira, Mariana Leitão, Raquel Bouça-Machado, Linda Azevedo Kauppila, António P. Veloso, Rui M. Costa, Joaquim J. Ferreira, Ricardo Matias

**Affiliations:** 1 Kinetikos, Coimbra, Portugal; 2 LBMF, CIPER, Faculdade de Motricidade Humana, Universidade de Lisboa, Cruz Quebrada, Dafundo Portugal; 3 CNS - Campus Neurológico Sénior, Torres Vedras, Portugal; 4 Instituto de Medicina Molecular João Lobo Antunes, Lisbon, Portugal; 5 Department of Neurosciences and Mental Health, Neurology, Hospital de Santa Maria, Centro Hospitalar Universitário Lisboa Norte, Lisbon, Portugal; 6 Champalimaud Research, Champalimaud Centre for the Unknown, Lisbon, Portugal; 7 Departments of Neuroscience and Neurology, Zuckerman Mind Brain Behavior Institute, Columbia University, New York, USA; 8 Laboratory of Clinical Pharmacology and Therapeutics, Faculdade de Medicina, Universidade de Lisboa, Lisbon, Portugal; 9 Champalimaud Clinical Centre, Champalimaud Centre for the Unknown, Lisbon, Portugal; 10 Human Movement Analysis Lab, Escola Superior Saúde – Instituto Politécnico de Setúbal, Setúbal, Portugal

**Keywords:** Parkinson’s disease, Gait analysis, Pathological gait, Wearable devices, Smartphone, Kinematics

## Abstract

**Background:**

Gait impairments are among the most common and impactful symptoms of Parkinson’s disease (PD). Recent technological advances aim to quantify these impairments using low-cost wearable systems for use in either supervised clinical consultations or long-term unsupervised monitoring of gait in ecological environments. However, very few of these wearable systems have been validated comparatively to a criterion of established validity.

**Objective:**

We developed two movement analysis solutions (3D full-body kinematics based on inertial sensors, and a smartphone application) in which validity was assessed versus the optoelectronic criterion in a population of PD patients.

**Methods:**

Nineteen subjects with PD (7 female) participated in the study (age: 62 ± 12.27 years; disease duration: 6.39 ± 3.70 years; HY: 2 ± 0.23). Each participant underwent a gait analysis whilst barefoot, at a self-selected speed, for a distance of 3 times 10 m in a straight line, assessed simultaneously with all three systems.

**Results:**

Our results show excellent agreement between either solution and the optoelectronic criterion. Both systems differentiate between PD patients and healthy controls, and between PD patients in ON or OFF medication states (normal difference distributions pooled from published research in PD patients in ON and OFF states that included an age-matched healthy control group). Fair to high waveform similarity and mean absolute errors below the mean relative orientation accuracy of the equipment were found when comparing the angular kinematics between the full-body inertial sensor-based system and the optoelectronic criterion.

**Conclusions:**

We conclude that the presented solutions produce accurate results and can capture clinically relevant parameters using commodity wearable sensors or a simple smartphone. This validation will hopefully enable the adoption of these systems for supervised and unsupervised gait analysis in clinical practice and clinical trials.

## Background

Parkinson’s disease (PD) presents a multitude of motor and non-motor symptoms heterogeneous in expression and progression over time [1, 2]. Alterations in full-body kinematics are seen since early stages of the disease and are characterized by reduced velocity, step length, arm swing and smoothness, increased inter-limb asymmetry, impairments in complex locomotor tasks (e.g., turning) and reduced range of motion at several joints (e.g., shoulder), leading in later stages to an increase in double-support time and cadence, shuffling steps, freezing of gait and festination [3]. Changes in lower and upper body kinematics have shown to be potential markers of disease progression in the early stage (number of steps, total duration, harmonic ratios and arm swing asymmetry) and middle stage (stride time variability and stride regularity) disease progression [4, 5].

Motor symptoms of PD are commonly rated using the Movement Disorder Society Unified Parkinson’s Disease Rating Scale (MDS-UPDRS), which has guided therapeutic decisions for several decades [1]. This approach is subjective and time-consuming, limited in assessment repetition and lacking in quantitative outcomes [6]. Full-body kinematic analysis systems are needed to obtain a full and objective assessment of movement as a basis for individually tailored clinical decision making and prognostication.

For decades, optoelectronic tracking systems have been established as a criterion in quantitative movement analysis [7]. However, such equipment is expensive, restricted to a controlled and calibrated environment, requiring a complex setup phase [7–9]. Wearable inertial sensors (e.g., inertial measurement units – IMU) have been introduced into clinical practice due to their relatively low cost, lightweight and ease of use [7–9] and have been proven to be useful and feasible for kinematic analysis of PD gait [10, 11]. However, very few studies applying inertial sensor systems quantitatively describe the changes in full-body kinematics in PD gait [10–13].

The previously described motor symptom assessment methods all require an in-person consultation with the patient. In between such assessments, motor symptoms and their fluctuations are reported using patient diaries [14, 15]. The risk of poor adherence, limited time resolution and subjective nature of such information raises concerns regarding their accuracy and reliability [14, 15]. It is necessary to expand beyond conventional clinical assessments and aim for movement analysis in unsupervised, ecologically valid and patient-relevant environments to achieve a more accurate kinematic characterization.

Quantitative unsupervised movement analysis could capture symptom fluctuations and rare events in PD while minimizing the effects of supervision [11, 14]. This would require an affordable, user-friendly system with minimal impact on daily living [16, 17]. Smartphones are owned by 81% of adults in the United States [18], contain one or several embedded inertial sensors and have been validated as a platform for ecological movement analysis [16, 19–22]. Benefits in terms of healthcare accessibility, patient engagement and reduction of clinician workloads are also envisioned or already proven to emerge from the use of mobile health technologies [23]. However, many studies addressing movement analysis using smartphone sensors have requirements that do not reflect typical usage, reducing ecological validity, and validation of applications for gait analysis of PD patients is limited [19].

Attempting to address current issues of quantitative motion analysis in terms of cost, time consumption and influence of supervision, two methods of objective and quantitative gait analysis were developed and are presented in this study: a 3D full-body kinematics analysis system based on inertial sensors for supervised objective evaluations, and a smartphone application for characterization of gait-related motor symptoms of PD in daily living scenarios beyond clinical assessments. To assess the validity of these methods for clinical practice and clinical trials, this study aims to simultaneously assess the estimation of the agreement between each of these two methods of objective and quantitative gait analysis and the optoelectronic criterion in patients with PD.

## Methods

### Recruitment and eligibility

The study was approved by Campus Neurológico Sénior (CNS) Ethics Committee (Ref. 04/2019) and all participants gave their written informed consent in accordance with the Declaration of Helsinki. Study participants were recruited between July and October 2019 from the in- and outpatients of the CNS, a tertiary specialized movement disorders center. After providing written informed consent, subjects with a PD diagnosis were evaluated for eligibility for study enrolment. Eligibility criteria included the ability to understand the potential risks and benefits of the study, willingness and ability to provide written informed consent to participate in the study, PD diagnosis (according to MDS criteria [24]), ability to walk unassisted, perform the Timed Up and Go test in a normal pace and without assistance in less than 11,5 s (i.e. no fall risk) [25], in ON phase, and Hoehn & Yahr scale < III. Exclusion criteria were defined as cardiovascular, pulmonary or musculoskeletal conditions that could affect patients’ ability to participate in the study, inability to correctly respond to the assessment protocol according to the clinician’s judgment or lack of support from a caregiver for this purpose and permanent use of gait assistance. A certified rater performed the MDS-UPDRS on all subjects. A summary of the subjects’ clinical outcomes is shown in Table 1.

**Table 1:** Summary of the clinical outcomes: Single-task Timed Up and Go test (TUG), and under Cognitive (CDT) and Motor (MDT) Dual-task, Movement Disorders Society - Unified Parkinson’s Disease Rating Scale (MDS-UPDRS) Total and Part III scores, Mini Balance Evaluation Systems Test (Mini-BESTest)

Test	Mean ± Standard deviation
TUG (s)	8.81 ± 1.52
TUG + CDT (s)	10.94 ± 3.05
TUG + MDT (s)	9.88 ± 2.12
MDS-UPDRS total	47.17 ± 20.01
MDS-UPDRS III	25.39 ± 11.29
Mini-BESTest	24.11 ± 3.02

A unique identification number was attributed to each participant enrolled in the study to maintain confidentiality.

### Experimental setup

Participants underwent a gait analysis barefoot, at a self-selected speed, for a distance of 10 m along the laboratory’s diagonal, going back and forth three times, never exceeding 1 min, and allowing the participant to rest whenever necessary. All participants received a careful explanation and were allowed a familiarization period in which they were given time to get accustomed to the equipment and space, and performed the task once whilst yet unrecorded. The participants’ motion during the walking trials was assessed simultaneously with an optoelectronic (used as the criterion), an inertial and a smartphone-based capture system.

Time synchronization between motion capture systems was achieved by sending a trigger signal from the optoelectronic system to the inertial system to start data capture.

For the optoelectronic system, 48 retroreflective markers were placed on the participants’ skin over specific anatomical landmarks (1st and 5th distal metatarsal head, *Hallux*’s distal phalanx, heel, medial and lateral malleoli, medial and lateral femoral condyles, anterior and posterior superior iliac spines, xiphoid process, jugular notch, spinous process of 7th cervical and thoracic vertebrae, acromion, medial and lateral humeral epicondyles, head of the ulna, styloid process of the radius, and distal phalanx of the middle finger). Additionally, four rigid clusters, each with four markers, were attached to the lateral sides of the thighs and shanks. The markers were placed by an experienced physiotherapist, always prior to placement of any IMU and were held in position with double-sided adhesive tape. The 3D coordinates of each marker were collected at 120 Hz by a system of 10 infrared cameras (Oqus 300+, Qualisys AB, Gothenburg, Sweden).

For the inertial system, fifteen IMU (Xsens, Enschede, Netherlands) with a sample rate set to 120 Hz, were placed and secured using elasticated velcro straps, in different body segments: head, thorax, scapulae, upper-arms, forearms, hands, sacrum, thighs, shanks and feet. Care was taken not to interfere with the reflective markers.

Two Nokia 5.1TM smartphones (Nokia, Espoo, Finland) were placed on the participants’ right and left front pockets, where Kinetikos CE-marked smartphone application (Kinetikos, Coimbra, Portugal) developed for Android (Google Inc., Mountain View, CA, USA) recorded linear and angular quantities captured by the device’s built-in hardware sensors throughout the trial (accelerometer, gyroscope, magnetometer and orientation data) at 100 Hz.

### Data analysis

Kinetikos CE-marked cloud-based platform (Kinetikos, Coimbra, Portugal) was used to reconstruct participants’ full-body motion using a 3D kinematic computer model of the skeletal system that includes representation of the head, thorax, upper and lower extremities and respective joints, totaling 26 degrees of freedom (DoF). In this model, the hip was a ball and socket joint (3 DoF). Both the knee and ankle had a single DoF each set by a revolute joint (1 DoF + 1 DoF). Head and lumbar motion were each modelled as ball-and-socket joints (3 DoF + 3 DoF). Each upper extremity consisted of 5 DoF; the shoulder was modelled as a ball-and-socket joint (3 DoF), and the elbow and forearm rotation were each modelled with revolute joints (1 DoF). Each joint’s coordinate system matches the International Society of Biomechanics (ISB) recommendations [26, 27]. Rotations were represented by Cardan-Euler angles, following the sequences proposed by the ISB [26, 27] for all joints except the shoulder, where the sequence XZY was chosen, which has shown superior results in terms of gimbal lock incidence and clinical coherence when compared to the ISB recommendation [28]. For this study, agreement between 11 DoF was considered: hip flexion/extension, hip adduction/abduction, hip internal/external rotation, knee flexion/extension, ankle dorsi/plantar flexion, arm flexion/extension, arm adduction/abduction, arm internal/ external rotation, elbow flexion/extension, forearm pronation/supination and wrist flexion/extension. Both virtual markers and virtual IMUs were placed on the 3D biomechanical model to match the locations of the experimental ones.

The markers’ 3D trajectories and IMU orientations were identified in Qualisys Track Manager (Qualisys AB, Gothenburg, Sweden) and Xsens MT Manager (Xsens, Enschede, Netherlands), respectively. The optoelectronic system’s markers’ trajectories were then exported while for each inertial sensor a file with inertial, angular and orientation data was generated. The model’s segments were proportionally scaled based on each participant’s height and the identified trajectories and orientations were expressed in the model’s coordinate system. While orientations from the inertial based system were centered around the pelvis by default, trajectories from the optoelectronic system were centered around the model’s pelvis by defining a coordinate system using the left and right anterior and posterior superior iliac spine markers [27] and expressing all marker trajectories in this system. Inverse kinematics was used to reconstruct the optimal body pose in each frame based on a global optimization procedure aiming to minimize the weighted sum of squared distances between measured and model-determined marker positions [29, 30]. This procedure was conducted, firstly, based on the optoelectronic data by minimizing the errors between experimental and model markers 3D coordinates, and secondly, on the IMUs data by minimizing the errors between the experimental IMU orientations and the model’s IMU Frames.

**Fig. 1:**
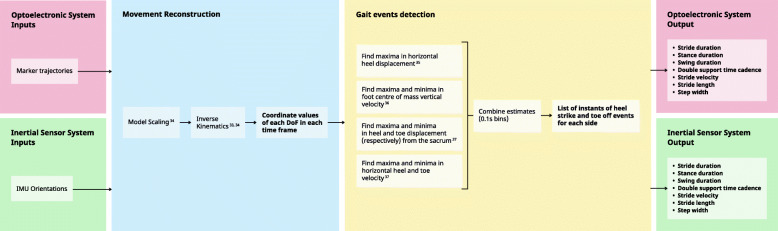
Schematic representation of the optoelectronic criterion and inertial sensor data analysis

The previously described method allows the model’s joint angles to be used to track the orientation of each body part. The results were two sets of coordinate values of the model’s DoF for each time frame of the gait trial. Gait events (left and right heel strike and toe-off events) were detected based on four kinematic-based procedures [31–33]. These algorithms use the calcaneus and toe marker displacement and velocity time series for the identification of: horizontal inter-heel distance [31]; foot center-of-mass vertical velocity [32]; horizontal heel and toe position relative to the pelvis; horizontal heel and toe velocity [33]. Since these estimates were found to identify heel strike and toe-off with unequal consistency [34], the event indices were further combined in a custom algorithm yielding a single, more robust outcome. Events with no correspondence on the optoelectronic system (i.e., events outside the area captured by the infrared cameras) were not considered for analysis. Because the camera system did not capture the beginning and end of the laboratory’s diagonal, gait events during turns were not captured by this system. This process is described in Fig. 1.

**Fig. 2:**
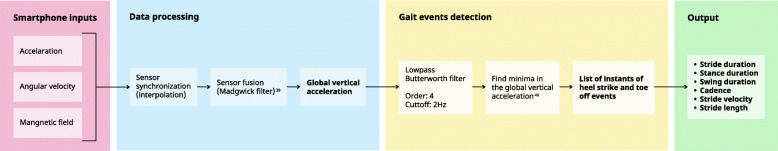
Schematic representation of the smartphone data analysis

Sensor synchronization in the Kinetikos CE-marked smartphone application was achieved through linear interpolation and spherical linear interpolation (for orientation data). Sensor fusion was performed using Madgwick’s gradient descent IMU orientation estimation, which combines attitude estimates by integration of gyroscope measurements and direction obtained by accelerometer measurements – to compensate for long term gyroscope integration drift – to obtain the device’s global orientation [35]. Gait events (heel strikes and toe offs ipsilateral to the side of the pocket the smartphone was placed in) were detected using a custom peak detection algorithm on the global vertical acceleration time series after filtering with a 4th order Butterworth lowpass filter at 2 Hz, based on the assessment that the vertical acceleration time series contains alternating high and low amplitude peaks and that the nadirs immediately following such peaks are correlated with heel-strike and toe-off events, respectively [36]. Only events from the mobile application with a corresponding event on the criterion were considered for analysis. This sequence of events is described in Fig. 2.

Several gait parameters were calculated for both systems: stride duration as the time difference between two ipsilateral heel-strikes; stance phase duration as the time between a heel-strike event and the following ipsilateral toe-off event; swing phase duration as the time between a toe-off event and the following ipsilateral heel-strike event; cadence as the number of strides per minute; stride length given by the difference in a foot’s position in the sagittal plane between consecutive ipsilateral heel-strikes for the inertial-based system and by the linear relation of stride length, stride frequency and acceleration variance [37] for the smartphone application; stride speed as stride length divided by stride time. In the case of the inertial sensor-based system, double-support phase duration and step width were also assessed, as the time between a heel-strike and the following contralateral toe-off and as the distance in the coronal plane between the two feet at the instant of a heel-strike, respectively.

### Statistical analysis

Bland-Altman analysis [38] was used to assess the agreement between the 3D full-body kinematics based on inertial sensors and the smartphone application, and the optoelectronic criterion. Differences between methods versus the average of their values (bias) and Limits of Agreement (LoA) were calculated along with 95% confidence intervals (CI). For each gait parameter, the assumption of normality of the differences between methods was checked with the D’Agostino-Pearson omnibus test. Bland-Altman analysis was performed with GraphPad Prism software (GraphPad Software, Inc., La Jolla, CA).

Welch’s two-sample t-test was used to analyze the significance of the differences in each gait parameter between the Normal difference distribution from PD patients versus healthy controls and from PD patients during ON versus OFF states and the bias of the inertial-based 3D full-body and the smartphone-based kinematics. Normal difference distributions were pooled from studies conducted in PD patients in ON and OFF states, that included an age-matched healthy control group [39–42].

Agreement between angular kinematics waveforms obtained from marker-based and IMU-driven inverse kinematics was assessed as follows: (i) average reconstruction error was taken as the mean absolute error (MAE); and (ii) waveform similarity was evaluated through the Linear Fit Method (LFM) [43]. The LFM yields three coefficients: α_1_, the amplitude scaling factor between the curves being compared and the reference time series; α_0_, which predicts any offset; and R^2^, the strength of the linear relation between the two time series. LoA for α_0_ and α_1_ were calculated to assess whether these parameters are statistically different from the same parameters of the line of equality. Concurrent validity was considered very high if R^2^ is above 0.75, fair-to-high if R^2^ is between 0.4–0.75, and low if R^2^ is below 0.4. The agreement between angular kinematics waveforms was performed in Python software (Python Language Reference, version 3.7, available at http://www.python.org).

## Results

### Demographic

Nineteen subjects with PD (7 female) were enrolled in the study (age: 62 ± 12.27 years; disease duration: 6.39 ± 3.70 years; HY: 2 ± 0.23), recruited from CNS, Torres Vedras, Portugal. Six hundred eighty five gait cycles were identified from the criterion system and compared to the corresponding gait cycles selected from the inertial sensor-based system and the mobile application. Due to data integrity problems, two subjects were excluded from the former validation and one from the latter.

### Agreement between methods

**Fig. 3:**
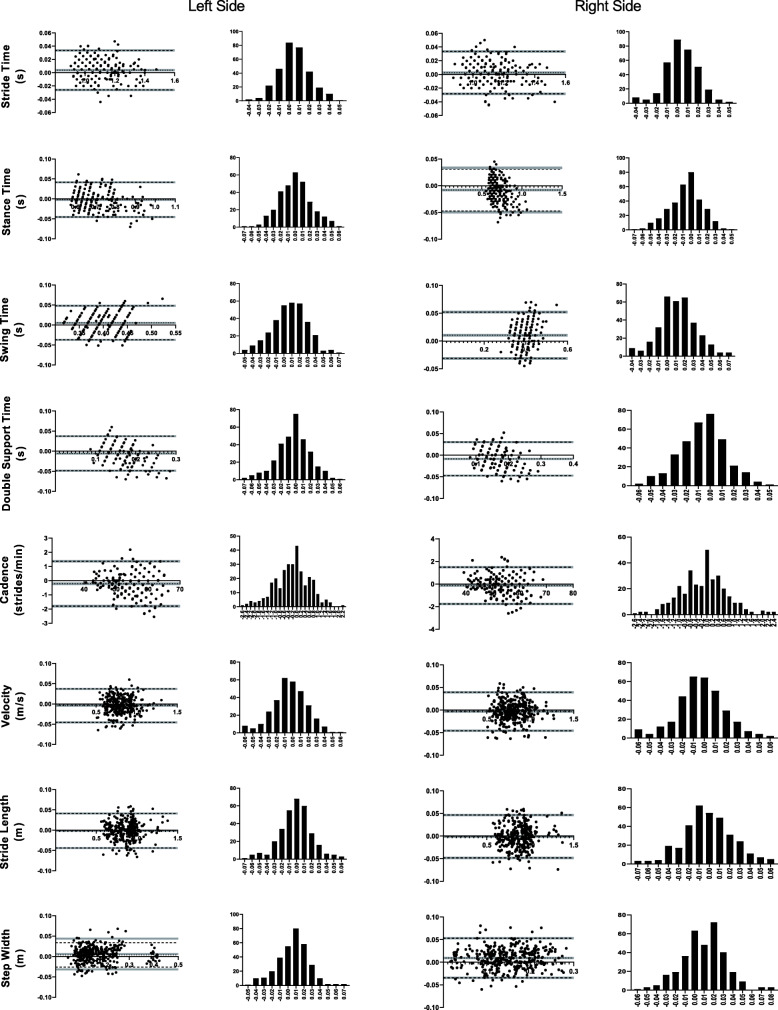
Bland-Altman analysis. Each gait parameter presents (for the left and ride side) a plot of differences between 3D full-body kinematics based on inertial sensors and the optoelectronic criterion versus the mean of the two measurements, and a distribution plot of differences between measurement by methods. Grey areas represent the 95% CI for bias and limits of agreement

**Fig. 4:**
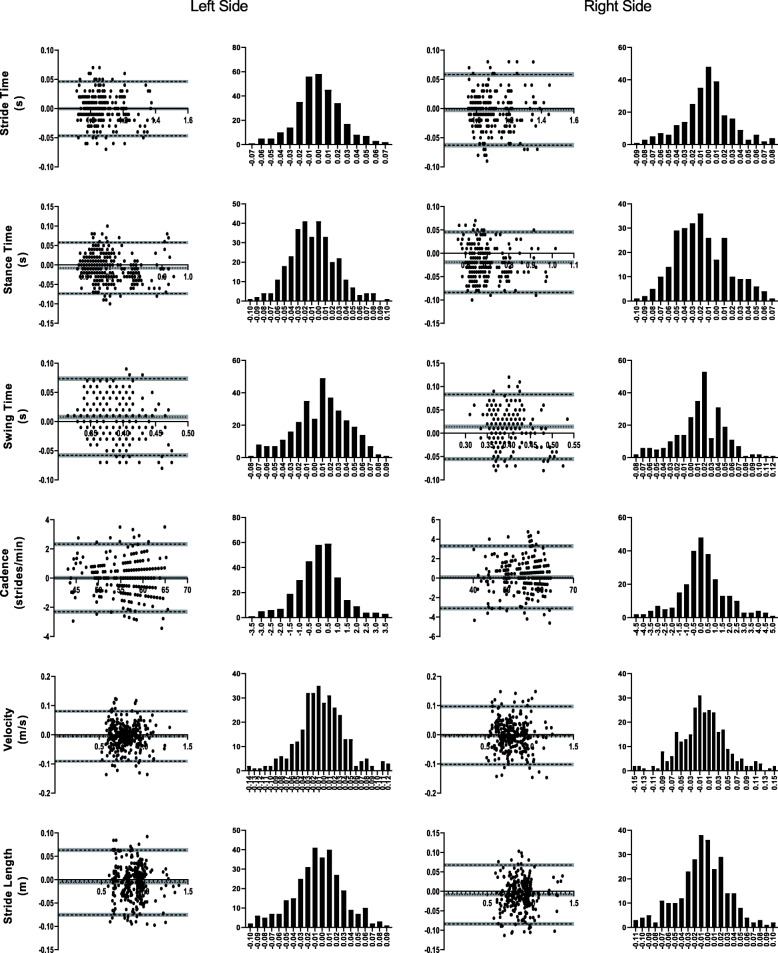
Bland-Altman analysis. Each gait parameter presents (for the left and ride side) a plot of differences between smartphone-based kinematics and the optoelectronic criterion versus the mean of the two measurements, and a distribution plot of differences between measurement by methods. Grey areas represent the 95% CI for bias and limits of agreement

Agreement of spatiotemporal parameters between either presented solution and the optoelectronic system used as a criterion was assessed through Bland-Altman analysis [38] (Figs. 3 and 4), resulting in an excellent agreement for all metrics. Bias for all spatiotemporal metrics was at least one order of magnitude below the mean values (Tables 2 and 3).

**Table 2:** Agreement between the gait parameters (mean, standard deviation and coefficient of variance - CV) measured by the inertial-based 3D full-body kinematics (IMU) and the optoelectronic criterion system (OS). Bias and Limits of Agreement (LoA) from the Bland-Altman analysis with 95% Confident Intervals (CI). The result of the D’Agostino-Pearson omnibus test (K2) is presented for each gait parameter with respective *p* value

		Mean ± Standard deviation		CV		Bias			LoA (lower)			LoA (upper)			D’Agostino & Pearson	
		*OS*	*IMU*	*OS (%)*	*IMU (%)*	*Bias*	*95% CI*	*95% CI*	*LoA*	*95% CI*	*95% CI*	*LoA*	*95% CI*	*95% CI*	*K2*	*p value*
Stride duration (s)	Left	1086 ± 0,112	1082 ± 0,113	10,293	10,445	0,004	0,002	0,006	−0,026	−0,028	−0,024	0,034	0,032	0,035	0,157	0,925
	Right	1092 ± 0,120	1089 ± 0,123	11,026	11,250	0,003	0,001	0,004	−0,028	−0,030	−0,027	0,034	0,032	0,035	3102	0,212
Stance duration (s)	Left	0,695 ± 0,084	0,6973 ± 0,088	12,037	12,592	−0,002	−0,005	0,000	−0,046	−0,048	− 0,043	0,042	0,039	0,044	0,148	0,929
	Right	0,692 ± 0,086	0,700 ± 0,094	12,426	13,460	−0,009	−0,010	− 0,006	− 0,047	−0,052	− 0,047	0,031	0,031	0,036	4681	0,096
Swing duration (s)	Left	0,391 ± 0,037	0,386 ± 0,034	9379	8861	0,005	0,003	0,008	−0,037	−0,039	− 0,035	0,048	0,046	0,050	1901	0,386
	Right	0,397 ± 0,040	0,386 ± 0,035	10,219	9065	0,010	0,008	0,013	−0,031	− 0,034	− 0,029	0,052	0,050	0,054	1299	0,522
Double support time (s)	Left	0,157 ± 0,029	0,162 ± 0,038	18,635	23,296	−0,006	− 0,008	− 0,003	− 0,049	−0,051	− 0,046	0,037	0,035	0,040	4525	0,104
	Right	0,143 ± 0,036	0,152 ± 0,015	25,192	27,339	−0,009	− 0,011	− 0,006	− 0,047	−0,050	− 0,045	0,030	0,028	0,032	0,218	0,897
Cadence (strides/min)	Left	55,788 ± 5321	56.003 ± 5410	9539	9660	− 0,215	-0,305	-0,124	− 1796	− 1887	− 1706	1366	1276	1457	2307	0,316
	Right	55,515 ± 5625	55,649 ± 5766	10,132	10,361	−0,134	− 0,224	− 0,044	− 1760	− 1851	− 1671	1492	1403	1583	3705	0,157
Stride speed (m/s)	Left	0,800 ± 0,134	0,804 ± 0,133	16,730	16,558	−0,004	− 0,006	− 0,002	−0,046	− 0,048	−0,043	0,037	0,035	0,040	3153	0,207
	Right	0,802 ± 0,142	0,805 ± 0,142	17,716	17,655	−0,003	− 0,006	− 0,001	− 0,046	−0,048	− 0,043	0,039	0,037	0,042	4451	0,108
Stride length (m)	Left	0,863 ± 0,141	0,864 ± 0,140	16,393	16,252	−0,001	− 0,004	0,001	− 0,044	−0,046	− 0,042	0,041	0,039	0,044	5230	0,073
	Right	0,868 ± 0,140	0,868 ± 0,138	16,114	15,884	−0,001	− 0,004	0,002	− 0,048	− 0,051	− 0,046	0,047	0,044	0,049	1173	0,556
Step width (m)	Left	0,172 ± 0,077	0,165 ± 0,076	45,050	46,051	0,006	0,004	0,008	−0,032	− 0,034	− 0,029	0,044	0,042	0,046	5416	0,067
	Right	0,153 ± 0,061	0,144 ± 0,059	39,942	41,241	0,009	0,007	0,012	−0,034	−0,037	−0,032	0,053	0,051	0,055	3812	0,149

**Table 3:** Agreement between the gait parameters (mean, standard deviation and coefficient of variance – CV) measured by the smartphone-based kinematics (Mob) and the optoelectronic criterion system (OS). Bias and Limits of Agreement (LoA) from the Bland-Altman analysis with 95% Confident Intervals (CI). The result of the D’Agostino-Pearson omnibus test (K2) is presented for each gait parameter with respective *p* value

		Mean ± Standard deviation		CV		Bias			LoA (lower)			LoA (upper)			D’Agostino & Pearson	
		*OS*	*Mob*	*OS (%)*	*Mob (%)*	*Bias*	*95% CI*	*95% CI*	*LoA*	*95% CI*	*95% CI*	*LoA*	*95% CI*	*95% CI*	*K2*	*p value*
Stride duration (s)	Left	1071 ± 0,098	1072 ± 0,100	9139	9375	0,000	−0,003	0,002	−0,047	−0,049	− 0,044	0,046	0,044	0,049	3480	0,176
	Right	1095 ± 0,109	1098 ± 0,109	9935	9947	−0,002	− 0,006	0,001	− 0,063	− 0,067	−0,059	0,058	0,054	0,062	3316	0,191
Stance duration (s)	Left	0,688 ± 0,079	0,696 ± 0,085	11,457	12,205	−0,008	−0,012	−0,004	− 0,074	−0,077	− 0,070	0,058	0,054	0,061	3969	0,137
	Right	0,686 ± 0,081	0,705 ± 0,085	11,878	12,045	−0,019	−0,023	− 0,015	− 0,084	−0,088	− 0,080	0,046	0,042	0,050	5530	0,063
Swing duration (s)	Left	0,387 ± 0,032	0,379 ± 0,037	8279	9747	0,008	0,004	0,012	−0,058	−0,062	− 0,054	0,074	0,070	0,077	4341	0,114
	Right	0,403 ± 0,039	0,389 ± 0,044	9661	11,303	0,014	0,010	0,018	−0,055	−0,060	− 0,051	0,083	0,079	0,087	5574	0,062
Stride speed (m/s)	Left	0,802 ± 0,136	0,807 ± 0,139	16,919	17,179	−0,005	−0,010	0,000	−0,091	−0,095	− 0,086	0,080	0,076	0,085	5921	0,052
	Right	0,808 ± 0,145	0,812 ± 0,148	17,951	18,244	0,014	−0,055	0,083	−0,102	− 0,108	−0,096	0,097	0,091	0,103	5246	0,073
Cadence (strides/min)	Left	56,303 ± 4882	56,287 ± 4983	8670	8852	0,016	−0,119	0,151	− 2303	− 2432	− 2162	2335	2194	2464	2681	0,262
	Right	54,984 ± 5347	54,885 ± 5325	9724	9701	0,099	− 0,100	0,299	− 3105	− 3295	− 2896	3303	3094	3494	4443	0,108
Stride length (m)	Left	0,862 ± 0,142	0,868 ± 0,145	16,512	16,731	−0,006	−0,010	− 0,002	−0,075	− 0,079	−0,072	0,063	0,059	0,067	1972	0,373
	Right	0,896 ± 0,121	0,906 ± 0,107	16,139	15,851	−0,008	−0,013	0,003	−0,084	−0,088	− 0,079	0,068	0,063	0,072	3317	0,190

### Resolution

To analyze the differences between the bias of either presented system and the Normal difference distribution from PD patients versus healthy controls and PD patients during ON and OFF medication states, Welch’s two-sample t-test was used, with normal difference distributions pooled from published research in PD patients in ON and OFF states that included age-matched healthy control groups [39–42]. As shown in Table 4, both gait analysis methods presented enough resolution to capture the differences between PD patients in the OFF phase and age-matched healthy controls, and between PD patients in OFF and ON medication states in all gait parameters with the exceptions of swing duration and step width.

**Table 4:** Absolute normal difference distribution (mean ± standard deviation) from PD patients versus healthy controls (PDvsControl) and Parkinson’s disease patients between ON and OFF states (ONvsOFF) for each gait parameter [39–42]. Bias (mean ± standard deviation) values for each gait parameter for the inertial-based 3D full-body kinematics (IMU Bias) and the smartphone-based kinematics (Mob Bias). *P-*value is determined by Welch’s *t-*test when comparing PDvsControl / IMU Bias, PDvsControl / Mob Bias, ONvsOFF / IMU Bias and ONvsOFF / Mob Bias

	PDvsControl *mean ± SD*	ONvsOFF *mean ± SD*	IMU Bias *mean ± SD*	Mob Bias *mean ± SD*	PDvsControl / IMU Bias *p value*	PDvsControl / Mob Bias *p value*	ONvsOFF / IMU Bias *p value*	ONvsOFF / Mob Bias *p value*
Stride duration (s)	0.040 ± 0.126	0.03 ± 0,156	0.003 ± 0,015	−0.001 ± 0,027	**0,0013**	**0,0014**	**0,0203**	**0,0166**
Stance duration (s)	0.044 ± 0,093	0.032 ± 0,094 ^a^	−0.005 ± 0.021	−0.014 ± 0,033	**< 0.0001**	**< 0.0001**	**0.0002**	**0.0002**
Swing duration (s)	0.009 ± 0,273	0.007 ± 0,053	0.008 ± 0,022	0.011 ± 0,034	0,9754	0,9518	0,8876	0,6776
Double support time (s)	0.253 ± 0.035 ^a^	0.164 ± 0,026 ^a^	−0.007 ± 0,021	–	**< 0,0001**	–	**< 0,0001**	–
Cadence (strides/min)	4.000 ± 16.101	3.500 ± 13.086	−0.175 ± 0,819	0.058 ± 1.405	**0,0331**	**0,0458**	**0,0077**	**0,0141**
Stride speed (m/s)	0.160 ± 0,325 ^a^	0.060 ± 0,030	−0.004 ± 0,021	−0.004 ± 0,047	**< 0,0001**	**< 0,0001**	**0,0040**	**0,0092**
Stride length (m)	0.210 ± 0,292 ^a^	0.140 ± 0,292 ^a^	−0.001 ± 0,023	−0.007 ± 0,037	**< 0,0001**	**< 0,0001**	**< 0,0001**	**< 0,0001**
Step width (m)	0.008 ± 0,031	0.001 ± 0,038	0.008 ± 0,021	–	> 0,9999	–	0,2520	–

a – indicates that statistical significant differences were reached in the original study from which the normal difference distribution was pooled. Significant results are displayed in bold

### Reconstruction error and waveform similarity

Average reconstruction error was taken as the MAE. MAE of 3091 ± 0,136° was achieved at the five lower limb DoF, while errors of 2348 ± 0,172° were found at the six upper extremity DoF. Waveform similarity, evaluated through the LFM [43], yielded mean coefficients of determination (R^2^) of 0,781 ± 0,050 for the lower DoF and 0,750 ± 0,022 for the upper DoF, indicating a very high waveform similarity. For the α_1_ (scaling factor) and α_0_ (scalar addition) parameters, respectively, mean values of 0.699 ± 0.350 and 2.606 ± 2.065° were achieved for the lower DoF and 0.671 ± 0.176 and 6.79 ± 4.199° for the upper DoF. Limits of agreement for the scaling factor and scalar addition were ± 0.685 and ± 4.046 for the lower DoF and ± 0.346 and ± 8.229 for the upper DoF.

**Fig. 5:**
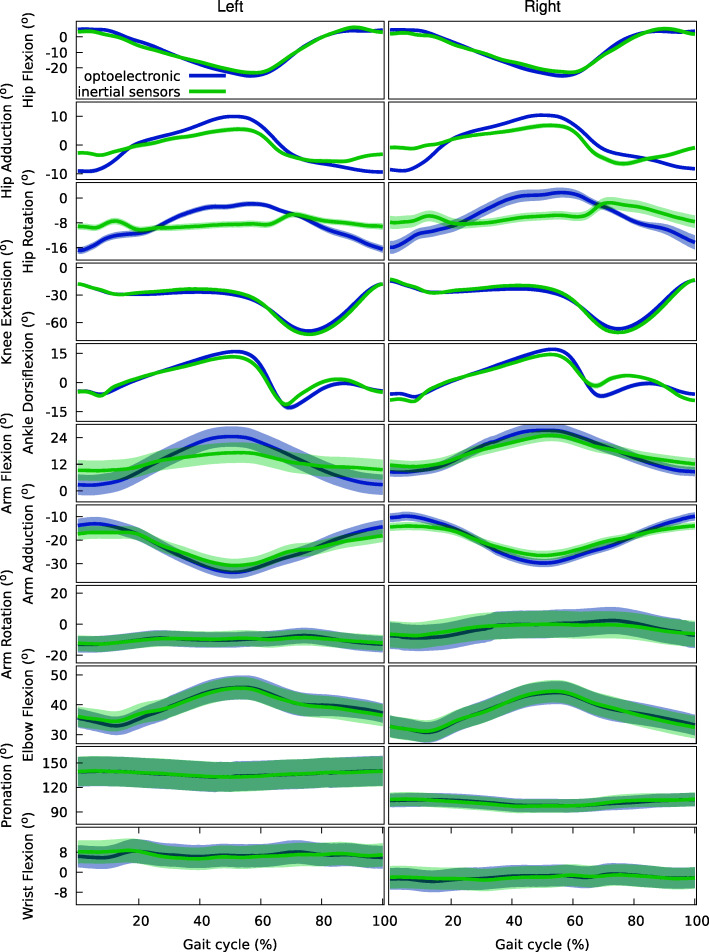
Joint angles throughout the normalized gait cycle for full-body kinematics based on inertial sensors (mean: solid green line: 95% confidence interval: green shaded areas) and the optoelectronic gold (mean: solid purple line: 95% confidence interval: purple shaded areas). Y-axis labels indicate positive values

These parameters indicate the validity of this method to support in-depth analysis of the full-body movement patterns of PD patients. More detailed results of the LFM are presented in Table 5. Figure 5 presents the mean and 95% confidence interval for joint angles throughout the normalized gait cycle, for the optoelectronic criterion and the inertial sensor-based system.

**Table 5:** Linear Fit Method [43] results comparing inertial-based 3D full-body kinematics and the optoelectronic criterion system. Outcome parameters are: α_1_ (scaling factor), α_0_ (scalar addition) and R^2^. Average reconstruction error is given by the Mean Absolute Error (MAE)

		Hip Flexion	Hip Adduction	Hip Rotation	Knee Extension	Ankle Dorsiflexion	Arm Flexion	Arm Adduction	Arm Rotation	Elbow Flexion	Pronation	Wrist Flexion
α_1_	Left	0,910	0,481	0,258	1117	0,816	0,395	0,653	0,622	0,668	0,933	0,593
	Right	0,903	0,487	0,035	1144	0,841	0,478	0,611	0,527	0,810	1040	0,725
α_0_	Left	− 1716	−0,467	− 6486	4331	0,051	7335	−11,419	− 3013	6777	− 8582	2687
	Right	− 3101	0,948	− 5370	− 2399	1187	−11,133	9456	− 0,108	7735	13,084	0,122
R^2^	Left	0,919	0,737	0,514	0,957	0,860	0,798	0,875	0,770	0,868	0,685	0,524
	Right	0,913	0,561	0,608	0,950	0,789	0,843	0,856	0,683	0,887	0,678	0,534
MAE (°)	Left	2296	3297	3511	2950	2924	2990	2170	2339	1830	2368	2342
	Right	2504	3630	3901	2957	2946	2713	2248	2504	2118	2615	1944

## Discussion

Gait impairments in PD can have a great impact on functional ability and quality of life and serve as indicators of global health, disease progression, cognition, fall risk and mortality [4, 5]. In this study, two methods for characterization of PD gait were developed and validated comparatively to the current optoelectronic criterion: a 3D full-body kinematic method using inertial sensors for use in clinical settings, and a mobile-based method to capture ecologically valid gait parameters.

Many relevant kinematics-related parameters of PD gait cannot be accurately captured through clinical observation in supervised in-office visits or subjective patient journals [6, 14, 15]. Limitations in conventional methods of motor symptom rating motivate the addition of quantitative and objective, supervised and unsupervised assessment strategies for effective and impactful clinical decisions, improving symptom ratings at in-office consultations and long-term, ecologically valid monitoring.

Wearable sensors have proven to be feasible instruments for accurate, quantitative movement analysis of PD patients in supervised environments, with advantages over the current optoelectronic systems in terms of cost, ease of use and environment requirements, although validation of full-body kinematic outputs using inertial sensor systems in PD patients is still lacking [10–13].

Current smartphones, with embedded inertial sensors, have shown potential and feasibility for unsupervised kinematic analysis during activities of daily living (ADL), effectively capturing relevant parameters [16]. Nonetheless, validation of smartphones’ use for movement analysis in PD patients is still limited [19–22].

### Agreement between methods

Bland Altman analysis for both the 3D full-body kinematics based on inertial sensors and the smartphone-based analysis shows an excellent agreement between the presented methods and the criterion.

Some assumptions need to be taken into consideration: the mean and standard deviation of the differences are constant throughout the range of measurements; and these are from an approximately normal distribution. Scatter plots and histograms of Figs. 3 and 4 bring light into each, respectively, showing the range of kinematic measurements follows a Gaussian distribution (verified by the D’Agostino-Pearson omnibus test results presented in Tables 2 and 3). Bias and LoA amplitude for all metrics was at least one order of magnitude below the mean values for each parameter, suggesting high agreement between the methods. The lack of observable trend in the differences across the measurements reinforces that there is a strong agreement between the systems across a range of measurements.

### Resolution

Either of the presented gait analysis methods showed enough resolution to capture the differences between PD patients and age-matched healthy controls, and PD patients’ differences between ON and OFF medication states in all gait parameters with exception of swing duration and step width – these differences were found to be non-statistically different in the original studies [39–42] (Table 4). These non-statistically significant differences are on the order of a thousandth of a second (swing duration) and thousandth of a meter (step width) which may raise a question about their clinical relevance.

### Reconstruction error and waveform similarity

Agreement between angular kinematics waveforms obtained from marker-based and IMU-driven inverse kinematics was assessed based on the MAE and the LFM. Average reconstruction error was estimated by the MAE, resulting in magnitudes inferior to the mean relative orientation accuracy of Xsens IMUs sensors of 5° for trials up to 60 s [44]. Results from the LFM showed high mean coefficients of determination, indicating a high waveform similarity across the full gait cycle for both lower- and upper-body DoF. The LoA of the scaling factor and scalar addition parameters of the LFM include 1 and 0, respectively, leading to conclude there exists a significant relationship between results obtained through the two movement analysis methods [45], albeit whether the LoA are small enough to ensure agreement requires a clinical perspective. The low MAE and high waveform similarity between the 3D full-body IMU-based movement reconstruction and optoelectronic criterion provide the needed evidence to support an in-depth analysis of the full-body movement patterns in PD patients using this method.

The previous points corroborate that the IMU-based gait analysis system is capable of producing results comparable to the criterion, providing a valid, objective full-body kinematic analysis with advantages in terms of cost, setup complexity and time consumption [7–9]. Nonetheless, this analysis is still subject to the effects of supervision and requires an in-person consultation. Complementing this approach, the smartphone-based solution, while unable to produce angular results or full-body gait analysis, has shown good agreement with the criterion and enough resolution to capture clinically relevant kinematic parameters, providing a quantitative assessment of spatiotemporal gait parameters with potential for use in ecologically valid environments with minimal impact on daily living, going beyond traditional, subjective measures of disease severity between consultations [14, 15]. The combination of these solutions allows for a more holistic, quantitative assessment of PD gait.

Further studies will be required to estimate the validity and reliability of these systems in a broader spectrum of the disease stage and respective motor symptoms. In addition, it is important to expand validation of the smartphone-based system beyond supervised environments and into patient-relevant scenarios to evaluate performance in unsupervised ADL involving more complex motions and providing more accurate insight into the patient’s motor symptoms.

## Conclusions

Our results show that the IMU-based full-body gait analysis provides accurate results using simple sensors with a reduced setup and environment complexity when compared to an optoelectronic system. The presented mobile application can be a user-friendly solution to collect ecologically valid gait parameters, translating into a greater clinical insight on previously unreported or subjectively reported motor symptoms. The resolution of either method is sufficient to capture relevant statistical differences in PD patients gait parameters when compared to age-matched controls, and between their ON and OFF states, making both presented systems clinically viable solutions with the potential to improve PD patients’ quality of life by opening the door to more personalized care based on quantitative objective metrics obtained not only during the moments of contact between patient and physician but longitudinally during ADL. Both the IMU-based and smartphone-based solutions produce results comparable to the criterion using commodity wearable technology. This validation will hopefully enable more widespread adoption of such systems for clinical practice and clinical trials.

## Data Availability

The datasets used and/or analyzed during the current study are available from the corresponding author on reasonable request.

## Abbreviations

ADL: Activities of Daily Living
CI: Confidence Interval
CNS: Campus Neurológico Sénior
DoF: Degrees of Freedom
IMU: Inertial Measurement Unit
ISB: International Society of Biomechanics
LFM: Linear Fit Method
LoA: Limits of Agreement
MDS: Movement Disorder Society
UPDRS: Unified Parkinson’s Disease Rating Scale
PD: Parkinson’s Disease

## References

[1] Maetzler W, Klucken J, Horne M (2016). A clinical view on the development of technology-based tools in managing Parkinson's disease. Mov Disord.

[2] Kalia L, Lang A (2015). Parkinson's disease. Lancet.

[3] Mirelman A, Bonato P, Camicioli R, Ellis T, Giladi N, Hamilton J (2019). Gait impairments in Parkinson's disease. The Lancet Neurology.

[4] Micó-Amigo M, Kingma I, Heinzel S, Rispens S, Heger T, Nussbaum S, et al. Potential markers of progression in idiopathic Parkinson’s disease derived from assessment of circular gait with a single body-fixed-sensor: a 5 year longitudinal study. Front Hum Neurosci. 2019;13. 10.3389/fnhum.2019.00059. 10.3389/fnhum.2019.00059 PMC6389786 30837857

[5] Belghali M, Chastan N, Cignetti F, Davenne D, Decker L (2017). Loss of gait control assessed by cognitive-motor dual-tasks: pros and cons in detecting people at risk of developing Alzheimer’s and Parkinson’s diseases. GeroScience..

[6] Maetzler W, Domingos J, Srulijes K, Ferreira J, Bloem B (2013). Quantitative wearable sensors for objective assessment of Parkinson's disease. Mov Disord.

[7] Pham M, Elshehabi M, Haertner L, Del Din S, Srulijes K, Heger T (2017). Validation of a step detection algorithm during straight walking and turning in patients with Parkinson’s disease and older adults using an inertial measurement unit at the lower Back. Front Neurol.

[8] Bouça-Machado R, Jalles C, Guerreiro D, Pona-Ferreira F, Branco D, Guerreiro T, Matias R, Ferreira JJ (2020). Gait kinematic parameters in Parkinson’s disease: a systematic review. J Parkinsons Dis.

[9] Schlachetzki J, Barth J, Marxreiter F, Gossler J, Kohl Z, Reinfelder S (2017). Wearable sensors objectively measure gait parameters in Parkinson’s disease. PLoS One.

[10] Caldas R, Mundt M, Potthast W, Buarque de Lima Neto F, Markert B. A systematic review of gait analysis methods based on inertial sensors and adaptive algorithms. Gait & Posture. 2017;57:204–210. [11] Brognara L, Palumbo P, Grimm B, Palmerini L. Assessing Gait in Parkinson’s Disease Using Wearable Motion Sensors: A Systematic Review. Diseases. 2019;7(1):18. 10.3390/diseases7010018 PMC6473911 30764502

[11] Espay A, Bonato P, Nahab F, Maetzler W, Dean J, Klucken J (2016). Technology in Parkinson's disease: challenges and opportunities. Mov Disord.

[12] Lewek M, Poole R, Johnson J, Halawa O, Huang X (2010). Arm swing magnitude and asymmetry during gait in the early stages of Parkinson's disease. Gait & Posture.

[13] Buckley C, Galna B, Rochester L, Mazzà C (2019). Upper body accelerations as a biomarker of gait impairment in the early stages of Parkinson’s disease. Gait & Posture..

[14] Vizcarra J, Sánchez-Ferro Á, Maetzler W, Marsili L, Zavala L, Lang A (2019). The Parkinson's disease e-diary: developing a clinical and research tool for the digital age. Mov Disord.

[15] Erb MK, Karlin DR, Ho BK, Thomas KC, Parisi F, Vergara-Diaz GP, Daneault JF, Wacnik PW, Zhang H, Kangarloo T, Demanuele C, Brooks CR, Detheridge CN, Shaafi Kabiri N, Bhangu JS, Bonato P (2020). mHealth and wearable technology should replace motor diaries to track motor fluctuations in Parkinson’s disease. NPJ Digit Med.

[16] Silsupadol P, Teja K, Lugade V (2017). Reliability and validity of a smartphone-based assessment of gait parameters across walking speed and smartphone locations: body, bag, belt, hand, and pocket. Gait Posture..

[17] Yang M, Zheng H, Wang H, McClean S, Harris N (2012). Assessing the utility of smart mobile phones in gait pattern analysis. Health Technol (Berl).

[18] Demographics of mobile device ownership and adoption in the United States [Internet]. Pewresearch.org. 2019 [cited2020 December 19]. Available from: https://www.pewresearch.org/internet/fact-sheet/mobile/

[19] Linares-del Rey M, Vela-Desojo L, Cano-de la Cuerda R. Mobile phone applications in Parkinson’s disease: a systematic review. Neurol (Engl Ed). 2019;34(1):38–54. 10.1016/j.nrl.2017.03.006 28549757

[20] Nishiguchi S, Yamada M, Nagai K, Mori S, Kajiwara Y, Sonoda T, Yoshimura K, Yoshitomi H, Ito H, Okamoto K, Ito T, Muto S, Ishihara T, Aoyama T (2012). Reliability and validity of gait analysis by android-based smartphone. Telemed J E Health.

[21] Hammoud A, Duchêne J, Abou-Ghaida H, Mottet S, Goujon J-M, Hewson DJ (2015). Validation of a smartphone gait analysis system. IFMBE proceedings.

[22] Furrer M, Bichsel L, Niederer M, Baur H, Schmid S (2015). Validation of a smartphone-based measurement tool for the quantification of level walking. Gait Posture.

[23] Postolache G, Postolache O (2019). Smartphone sensing technologies for tailored Parkinson’s disease diagnosis and monitoring. Mobile solutions and their usefulness in everyday life.

[24] Postuma RB, Berg D, Stern M, Poewe W, Olanow CW, Oertel W, Obeso J, Marek K, Litvan I, Lang AE, Halliday G, Goetz CG, Gasser T, Dubois B, Chan P, Bloem BR, Adler CH, Deuschl G (2015). MDS clinical diagnostic criteria for Parkinson’s disease. Mov Disord.

[25] Nocera JR, Stegemöller EL, Malaty IA, Okun MS, Marsiske M, Hass CJ (2013). Using the timed up & go test in a clinical setting to predict falling in parkinson’s disease. Arch Phys Med Rehabil.

[26] Wu G, van der Helm F, Veeger HE, Makhsous M, van Roy P, Anglin C, Nagels J, Karduna AR, McQuade K, Wang X, Werner FW, Buchholz B, International Society of Biomechanics (2005). ISB recommendation on definitions of joint coordinate systems of various joints for the reporting of human joint motion - part II: shoulder, elbow, wrist and hand. J Biomech.

[27] Wu G, Siegler S, Allard P, Kirtley C, Leardini A, Rosenbaum D, Whittle M, D'Lima DD, Cristofolini L, Witte H, Schmid O, Stokes I, Standardization and Terminology Committee of the International Society of Biomechanics (2002). ISB recommendation on definitions of joint coordinate system of various joints for the reporting of human joint motion - part I: ankle, hip, and spine. J Biomech.

[28] Senk M, Chèze L. Rotation sequence as an important factor in shoulder kinematics. Clin Biomech (Bristol, Avon). 2006;21 Suppl 1:S3–8. 10.1016/j.clinbiomech.2005.09.007 16274906

[29] Lu TW, O’Connor JJ (1999). Bone position estimation from skin marker co-ordinates using global optimisation with joint constraints. J Biomech.

[30] Delp SL, Anderson FC, Arnold AS, Loan P, Habib A, John CT, Guendelman E, Thelen DG (2007). OpenSim: open-source software to create and analyze dynamic simulations of movement. IEEE Trans Biomed Eng.

[31] Banks JJ, Chang W-R, Xu X, Chang C-C (2015). Using horizontal heel displacement to identify heel strike instants in normal gait. Gait Posture..

[32] O’Connor CM, Thorpe SK, O’Malley MJ, Vaughan CL (2007). Automatic detection of gait events using kinematic data. Gait Posture..

[33] Zeni JA, Richards JG, Higginson JS (2008). Two simple methods for determining gait events during treadmill and overground walking using kinematic data. Gait Posture..

[34] Hendershot BD, Mahon CE, Pruziner AL (2016). A comparison of kinematic-based gait event detection methods in a self-paced treadmill application. J Biomech.

[35] Madgwick SOH, Harrison AJL, Vaidyanathan A (2011). Estimation of IMU and MARG orientation using a gradient descent algorithm. IEEE Int Conf Rehabil Robot.

[36] Manor B, Yu W, Zhu H, Harrison R, Lo O-Y, Lipsitz L, Travison T, Pascual-Leone A, Zhou J (2018). Smartphone app–based assessment of gait during normal and dual-task walking: demonstration of validity and reliability. JMIR MHealth UHealth.

[37] Ladetto Q (2000). On foot navigation: continuous step calibration using both complementary recursive prediction and adaptive Kalman filtering. Ion Gps.

[38] Altman, DG. & Bland, JM. (1983) Measurement in medicine: the analysis of method comparison studies. Stat. 1983;32:307.

[39] Rafferty MR, Prodoehl J, Robichaud JA, David FJ, Poon C, Goelz LC, Vaillancourt DE, Kohrt WM, Comella CL, Corcos DM (2017). Effects of 2 years of exercise on gait impairment in people with Parkinson disease: the PRET-PD randomized trial. J Neurol Phys Ther.

[40] Beck Y, Herman T, Brozgol M, Giladi N, Mirelman A, Hausdorff JM. SPARC: a new approach to quantifying gait smoothness in patients with Parkinson’s disease. J Neuroeng Rehabil [Internet]. 2018;15(1). Available from: 10.1186/s12984-018-0398-3 10.1186/s12984-018-0398-3 PMC6006701 29914518

[41] Gilmore G, Gouelle A, Adamson MB, Pieterman M, Jog M (2019). Forward and backward walking in Parkinson disease: a factor analysis. Gait Posture..

[42] Salarian A, Russmann H, Vingerhoets FJG, Dehollain C, Blanc Y, Burkhard PR, Aminian K (2004). Gait assessment in Parkinson’s disease: toward an ambulatory system for long-term monitoring. IEEE Trans Biomed Eng.

[43] Iosa M, Peppe A, Morone G, Bottino S, Bini F, Marinozzi F, Paolucci S (2018). Assessment of waveform similarity in electromyographical clinical gait data: the linear fit method. J Med Biol Eng.

[44] Lebel K, Boissy P, Hamel M, Duval C (2015). Inertial measures of motion for clinical biomechanics: comparative assessment of accuracy under controlled conditions - changes in accuracy over time. PLoS One.

[45] Twomey PJ, Kroll MH (2008). How to use linear regression and correlation in quantitative method comparison studies: linear regression in method comparison studies. Int J Clin Pract.

